# On the effectiveness of graph matching attacks against privacy-preserving record linkage

**DOI:** 10.1371/journal.pone.0267893

**Published:** 2022-09-22

**Authors:** Youzhe Heng, Frederik Armknecht, Yanling Chen, Rainer Schnell

**Affiliations:** 1 School of Business Informatics and Mathematics, University of Mannheim, Mannheim, Baden-Württemberg, Germany; 2 Research Methodology Group, University of Duisburg-Essen, Duisburg, Nordrhein-Westfalen, Germany; King Saud University, SAUDI ARABIA

## Abstract

Linking several databases containing information on the same person is an essential step of many data workflows. Due to the potential sensitivity of the data, the identity of the persons should be kept private. Privacy-Preserving Record-Linkage (PPRL) techniques have been developed to link persons despite errors in the identifiers used to link the databases without violating their privacy. The basic approach is to use encoded quasi-identifiers instead of plain quasi-identifiers for making the linkage decision. Ideally, the encoded quasi-identifiers should prevent re-identification but still allow for a good linkage quality. While several PPRL techniques have been proposed so far, Bloom filter-based PPRL schemes (BF-PPRL) are among the most popular due to their scalability. However, a recently proposed attack on BF-PPRL based on graph similarities seems to allow individuals’ re-identification from encoded quasi-identifiers. Therefore, the graph matching attack is widely considered a serious threat to many PPRL-approaches and leads to the situation that BF-PPRL schemes are rejected as being insecure. In this work, we argue that this view is not fully justified. We show by experiments that the success of graph matching attacks requires a high overlap between encoded and plain records used for the attack. As soon as this condition is not fulfilled, the success rate sharply decreases and renders the attacks hardly effective. This necessary condition does severely limit the applicability of these attacks in practice and also allows for simple but effective countermeasures.

## Introduction

For many research problems, linking records on the same person from different databases is required [1]. An example would be the linkage of health information from different hospitals and death registries. If no unique personal identification number is available in all databases involved in the linkage, error-prone quasi-identifiers such as names, dates of birth, or addresses have to be used to identify a person.

However, records may contain sensitive information; therefore, the quasi-identifiers used for linkage should not be revealed to other persons, including the owners of the other databases. Privacy-preserving techniques are becoming more and more important in many real-world applications. Private cloud services and telecare medical information system(TMIS) are two examples where users’ privacy is considered as a vital aspect when designing such systems [2, 3].

Privacy-preserving record linkage (PPRL) is the research area driven by the need of linking sensitive data while not revealing the quasi-identifiers of the persons in the databases. These rely on shifting the linkage process from plain (quasi-)identifiers to encoded (quasi-)identifiers.

The past decade has seen a sharp increase in research on PPRL methods. PPRL techniques are usually classified as either secure multi-party computation (SMC) based or perturbation based [4]. SMC based techniques are provably secure and accurate but impose high communication and computation costs. Therefore perturbation based techniques are mostly considered better suited for real-world applications.

Due to the efficiency, Bloom filter based PPRL schemes (BF-PPRL) are among the most popular perturbation based PPRL techniques and have been used in practice [5, 6]. They deploy an encoding technique that derives a kind of fingerprint of a set such that set membership can be tested for given elements (up to some probability). In a nutshell, a BF-PPRL first expresses the quasi-identifiers as a set and then computes its Bloom filter.

However, researchers have shown that it can be attacked by different approaches. The most successful attacks so far are pattern mining attacks [7] and graph matching attacks [8]. While there exist several ideas for protecting against pattern mining attacks [9, 10], no countermeasures are known for graph matching attacks. Hence, these attacks are commonly considered to be a serious threat to PPRL.

The idea of using graph matching for attacks on BF-PPRL was proposed in [11], which showed that encoded names could be re-identified accurately under the assumption of complete knowledge of how a string is encoded. In the same year, [12] presented another attack on a different encoding (keyed-hash message authentication code, HMAC) scheme using subgraph matching. The attack was targeted on the PPRL methods used by the Office of National Statistics in the UK (ONS).

In [8], the authors reported the results of graph matching attacks on different databases. In the experimental evaluation of the attacks, almost all encoded records were correctly identified. However, their experiments considered a setting where an attacker has access to the encoded database *and* the corresponding plaintext database. Hence, one could say that the attack boiled down to linking these records of two copies of the same database—one given in plaintext, the other being encoded. Even though one can assume that in practice an attacker may have access to a plaintext database (for instance a town registry) that is related to the encoded database, most likely it will not be identical (in the sense of what individuals are stored). In most settings, the population covered by the encoded database of interest (for example, a medical registry) will not be the same population covered by the available plaintext database (for example, an insurance database). In the best case, an attacker may hope that the population of the encoded database is a subset of the population covered by the plaintext database. In general, even this may not be true. Although the intersection of their sets of records might be substantial, their set of different records, i.e., records that appear in one database only, will not be empty.

In this paper, the effect of the size of the overlap between the databases used for the attack on the attack’s success is systematically studied. We show that with decreasing overlap, the success probability sharply decreases. Furthermore, we discuss one method to harden PPRL schemes against graph matching attacks. We conclude that while graph matching represents without doubt an important attack method, it should not be used as an argument against the application of PPRL techniques in practice.

## Privacy-preserving record linkage

Record linkage refers to the task of identifying records in different data sets, containing different information on the same individual. Being already a challenge by itself, the situation becomes even more challenging when privacy regulations need to be respected. That is, such regulations usually forbid exposing records of data sets to other parties.

Privacy-Preserving Record Linkage (PPRL) schemes aim to link records of individuals from different databases such that a re-identification of the individuals is not possible for the linking party. The process is initiated by a data analyst C, who aims to conduct an analysis of certain features of individuals. To this end, it intends to involve (data of) records stored in some external databases, e.g., the database of patients of a hospital. For privacy reasons, it is not acceptable to send the full records to C as this would allow for direct identification of the individuals. Therefore, one assumes that records can be divided into two parts: linkage data and microdata. Linkage data refers to identifiers such as name, birthday, etc., i.e., information that may be used to re-identify individuals.

To keep the following description simple, we focus on the case of two databases (the extension of the description to ≥3 databases is straightforward). That is, we consider two database holders *A* and *B*. Each database holder is in possession of a data set of records. Here, we assume that any record *rec* can be represented as

$$\begin{array}{c} rec=(\text{ID},\lambda ,\mu ) \end{array}$$ (1)

where ID is some randomly chosen, unique identifier, λ is the linkage data such as name that will be used for linking records, and *μ* is the microdata that is relevant for the analysis.

Following the so-called separation principle, we assume a fourth party, the linkage unit L, who is responsible only for linking the records. In the separation principle, each party in a linkage protocol does only have access to the data it needs to perform its role in the protocol. That is, those parties involved in the actual linkage of records (here: L) can only see the quasi-identifiers, while those involved in the analysis of the linked data (here: C) only obtain microdata without direct identifiers. That is, L only gets access (indirectly) to the linkage data while the data analyst C gets only access to the microdata.

PPRL uses an encoding algorithm that transforms the plain linkage data λ into an encoded linkage data, referred to as [λ]. Database holder *A* applies this algorithm to the linkage data of any record stored in its database and sends the encoded linkage data to the linkage unit L. That is, given a record $rec_{A}=\left({\text{ID}}_{A},\lambda_{A},\mu_{A}\right)$, the linkage data λ_*A*_ is encoded into [λ_*A*_] and the tuple $\left({\text{ID}}_{A},[\lambda_{A}]\right)$ is sent to L. In parallel, the tuple $\left({\text{ID}}_{A},\mu_{A}\right)$ is sent to the data analyst C. Database owner *B* proceeds analogously.

**Fig 1:**
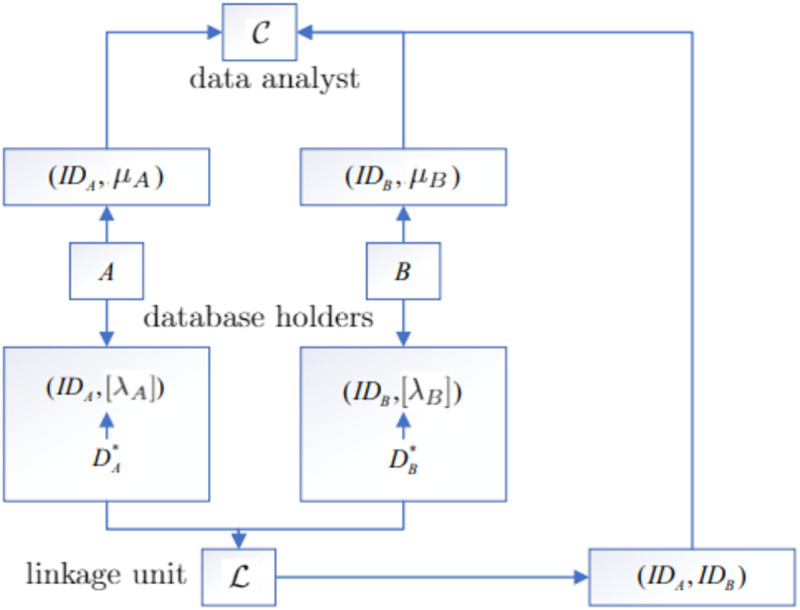
Privacy-preserving record linkage process.

Given these tuples, the linkage unit L starts the linkage process by executing the linkage algorithm on pairs (${\text{ID}}_{A}$,[λ_*A*_]) and (${\text{ID}}_{B}$,[λ_*B*_]). If the algorithm flags the pair as to be linked, the IDs $\left({\text{ID}}_{A},{\text{ID}}_{B}\right)$ are sent to C. The data analyst can now lookup in his internal data the entries $\left({\text{ID}}_{A},\mu_{A}\right)$ and $\left({\text{ID}}_{B},\mu_{B}\right)$ and can conclude that with high probability, the two sets of microdata stem from the same individual. The whole process is shown in Fig 1.

The linkage algorithm is based on two similarity functions: sim and ${\text{sim}}^{*}$. sim is the similarity function applied to pairs of linkage data in plaintext and ${\text{sim}}^{*}$ on pairs of encoded linkage data. The record linkage scheme decides that two records (ID,λ,μ) and $\left({\text{ID}}^{\prime },\lambda^{\prime },\mu^{\prime }\right)$ should be linked if $\text{sim}(\lambda ,\lambda^{\prime })$ is above a certain threshold. Likewise, in a PPRL the linkage process suggests that two records should be linked if ${\text{sim}}^{*}\left([\lambda ],[\lambda^{\prime }]\right)$ is sufficiently large. For example, BF-PPRL has been suggested with Dice similarity for the sim and the Hamming distance for ${\text{sim}}^{*}$.

PPRL schemes need to be effective, correct, and secure. Effective means that the effort of encoding and linkage should scale well with the data set sizes.

Correct means that the linkage process executed on the encoded records should come to the same results (with high probability) as if executed on the plain records. In other words, the linkage process executed on [λ_*A*_] and [λ_*B*_] should suggest that the underlying records *rec*_*A*_ and *rec*_*B*_ should be linked if and only if the underlying plain records *rec*_*A*_ and *rec*_*B*_ refer to the same individual with high probability.

Finally, secure means that it should not be possible to re-identify individuals from the encoded records. To this end, one assumes that all parties *A*, *B*, L, and C are *honest-but-curious*. This means that each party faithfully executes the protocols but may analyze the data to learn information about individuals represented in the data sets that are not under control. Moreover, one cannot exclude that certain meta-information about the data sets are known, e.g., that they belong to a specific hospital, the individuals are residents of a certain area, etc.

## Graph matching attack

**Fig 2:**
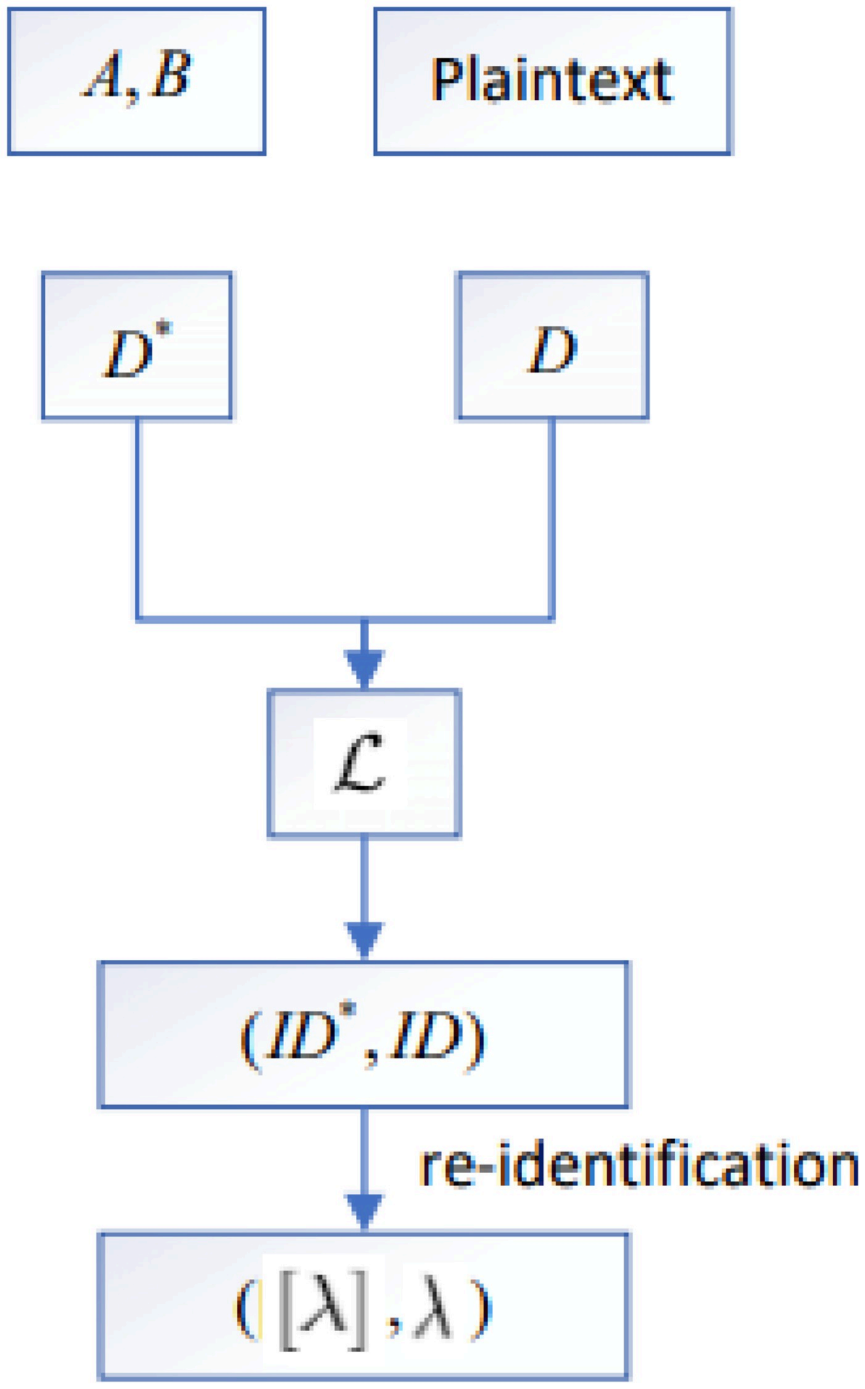
Graph matching attack description.

A graph matching attack [8] considers a scenario as shown in Fig 2. The attacker has access to a plaintext database *D* and an encoded database D*. The attacker’s goal is to identify elements in D* that can be linked to records in *D*. As the latter are given in plaintext, this would result into a re-identification of the individual encoded in D*. Such scenario is motivated by the fact that quite often, an attacker has some information about a super-set of the records contained in D*. For example, D* may contain patient records from a hospital while *D* may be the publicly available phone book.

Going into more technical details, the plaintext database *D* contains several records *rec* = (λ, *μ*) while D* is a collection of encoded linkage data [λ]. The latter are bitstrings, being the Bloom filters computed from λ. To simplify the following descriptions, we will write λ ∈ *D* to express that *D* contains a record (λ, *μ*) and likewise [λ] ∈ D*. Moreover, as the encoding process is usually deterministic (once the parameters are fixed), we will write λ ∈ D* to express that [λ] ∈ D*.

For linking λ ∈ *D* and [λ] ∈ D*, the attacker exploits the fact that many existing PPRL schemes use an encoding scheme that preserves the level of similarity. That is, it holds for any pair of plaintext records (*rec*, *rec*′) that $\text{sim}(rec,rec^{\prime })$ scales with ${\text{sim}}^{*}\left([rec],[rec^{\prime }]\right)$ and vice versa. Thus, if several records in *D* are also contained in D* (in encoded form), the relation between them in terms of similarity should have a similar structure in both databases. For example, if three records *rec*_*i*_ = (λ_*i*_, *μ*_*i*_)∈*D*, *i* = 1, 2, 3, are considered to be similar with respect to sim, then the encoded records [λ_1_], [λ_2_], [λ_3_] are likewise rather similar with respect to ${\text{sim}}^{*}$. If a fourth record *rec*_4_ = (λ_4_, *μ*_4_) has a low similarity to *rec*_1_, *rec*_2_, and *rec*_3_, then this holds also for [λ_4_] with respect to [λ_1_], [λ_2_], [λ_3_], and so on. In a graph matching attack, these information are encoded into so-called similarity graphs.

**Fig 3:**
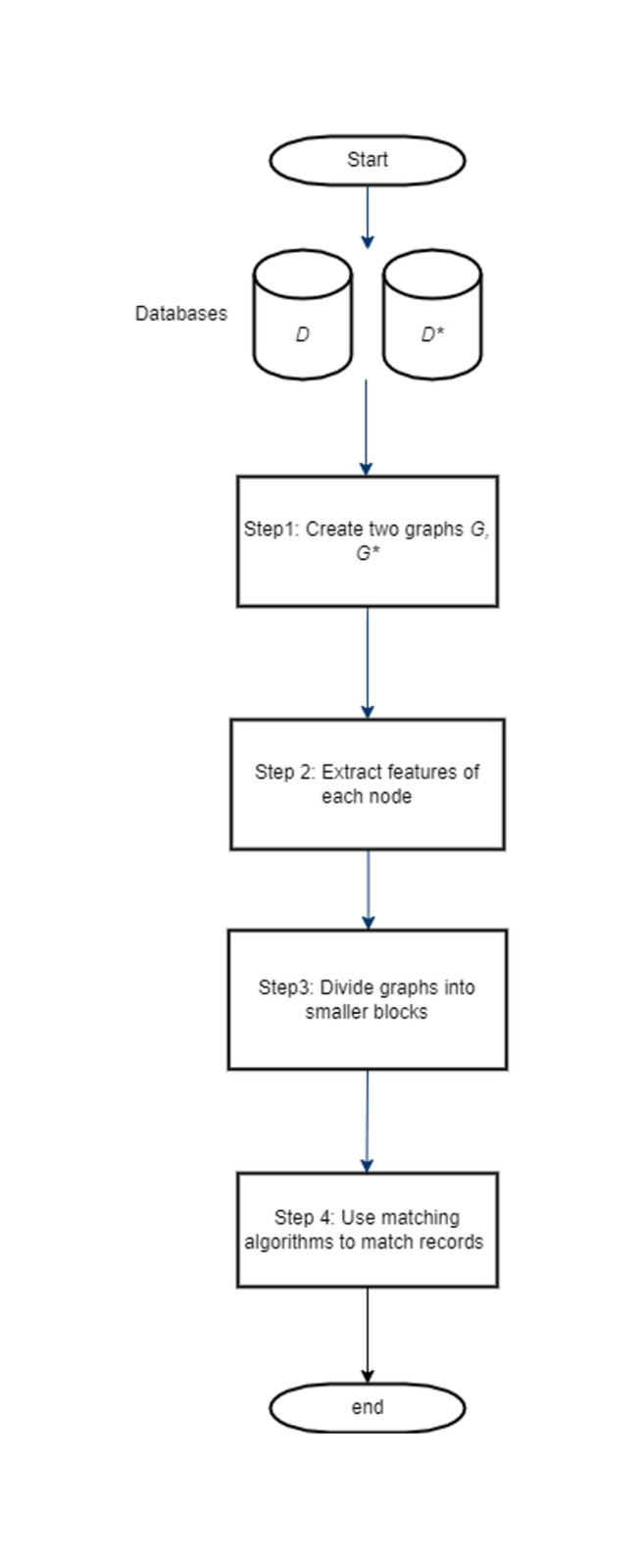
Flowchart of graph matching attack.

The graph matching attack is composed of four steps, as shown in Fig 3. The concrete working principle of these steps is not relevant for our analysis. Therefore, we explain the main ideas for each step only and refer to [8] for a detailed description.

In the first step, two similarity graphs *G* and G* are constructed—one over *D* using sim and one over D* using ${\text{sim}}^{*}$. Recall that a graph is defined by two sets, the set of vertices and the set of edges where each edge connects two vertices. For the first graph *G* = (*V*, *E*), the set of vertices is defined as *V* = {λ|λ ∈ *D*}. Moreover, edges are defined for any pairs λ, λ′ ∈ *D* with λ ≠ λ′ and $\text{sim}(\lambda ,\lambda^{\prime })$ being above some threshold value *τ*. That is, a record is connected to other records (within *G*) only if they are sufficiently similar. Moreover, any edge (λ, λ′)∈*E* is labelled with the similarity score $\text{sim}(\lambda ,\lambda^{\prime })$ of the vertices it connects. G* = (V*, E*) is defined analogously.

Recall that the basic idea is that if some [λ′] ∈ V* is the encoding of some λ ∈ *V*, their levels of similarity to neighbouring nodes should have a similar structure. The task of step two in the attack is to represent the considered structure of the neighbourhood by a feature vector. The considered features of the node are divided into three categories, namely node based, edge based, and structural based. Naturally, the list of features is different for the vertices in *G* and G*. Table 1 lists a selection of features from which the feature vector for each node is generated. One example is the ‘Length’ feature. The ‘Length’ of a node λ in *G* is the number of *q*-grams in λ, while the ‘Length’ of a node [λ] in G* is the number of 1-bits in [λ]. The ‘Max. Sim’ of λ is the maximum value of the edges connected to λ. We refer to [8] for a full, detailed list of the considered features.

**Table 1:** Features for nodes (CC: Connected Component; Avg: Average; Std: Standard Deviation).

Node based	Edge based	Structural based
Frequency	Degree	CC Degree
Length	Max. Sim	CC Density
	Min. Sim	Betweenness Centrality
	Avg. Sim	Degree Centrality
	Std. Sim	…

A special case are nodes that are connected to only few other nodes (or no nodes at all, being so-called singletons). For these, the features are not sufficiently characteristic to be helpful for the attack. Therefore, these kind of nodes are discarded and not considered anymore in the next steps.

The aim for steps three and four is to match similar structures from both graphs. To accelerate the attack, in step three the nodes are divided into smaller group so that the search for matches can be restricted to those. Finally, step four applies some graph matching algorithm to match vertices from the two graphs. Examples of the algorithms are symmetric highest matching, Hungarian matching, and stable marriage algorithms. Symmetric highest matching is to choose edges with highest similarities for both of the nodes. Hungarian algorithm aims for finding the minimum cost based assignments for a given connected component. Stable marriage algorithm is to find the most stable edges throughout the graph.

## Experimental evaluation of graph matching attacks

### Overlap rate

In [8], the authors showed that graph matching attacks can be highly effective under the assumption that for any true matches, the corresponding nodes in *G* and G*, respectively, are the center of similar sub-graphs. True matches are record pairs from *D*×D* where the linkage data are sufficiently similar (and hence where the records probably refer to the same person). We refer by $\text{Matches}(D,D^{*})$ to the set of true matches. Note that true matches are the only pairs of records that should/can be linked. Thus, the larger the size of $\text{Matches}(D,D^{*})$ in comparison to the size of the databases, the higher the fraction of records that are re-identified.

To this end, the authors defined the notion of the *overlap rate*:

$$\begin{array}{c} OverlapRate=\frac{2\cdot |\text{Matches}(D,D^{*})|}{\left|D^{*}|+|D\right|} \end{array}$$ (2)

The overlap rate ranges from 0% to 100%, the latter being the case if *D* equals D*. In [8], it was demonstrated that graph matching attacks are very powerful if the overlap rate is *high*, i.e., very close to 100%. However, as this condition is rarely given in practice, it raises the following question: How do graph matching attacks perform if the overlap rate is not close to 100%?

To address this question, we conduct a number of experiments to evaluate the attack for a varying set of overlap rates. The parameter choices will be discussed below. Moreover, for each attack different parameter settings (about 80 in total) of different matching methods are tested. For each combination, the experiment is repeated five times and the maximum accuracy is reported.

**Fig 4:**
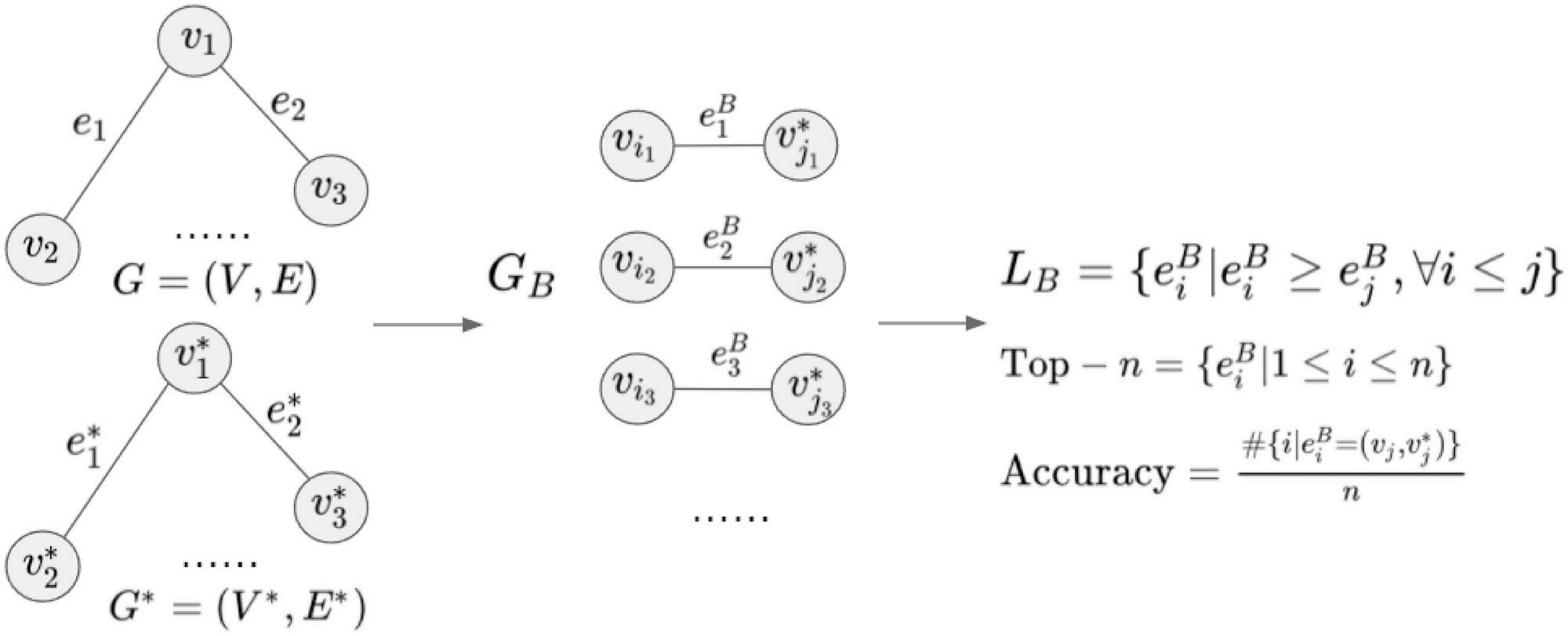
Result and evaluation of the attack. Each pair $\left(v_{j},v_{j}^{*}\right)$ refers to same individual in the example.

Here, the accuracy is determined as follows follows. For each parameter setting, a bi-partite graph *G*_*B*_ = (*V*, V*, *E*_*B*_) is generated. This means that each edge connects a node from *V* with a node from V*. Recall that the nodes in *V* and V* represent records in *D* and D*, respectively. The edges are labelled with a similarity confidence of the attack algorithm for each pair of records. The higher this value, the higher the probability that the considered pair represents a true match. Thus, a natural metric for the level of success is to how often high similarity confidence values refer to true matches. To this end, a list *L*_*B*_ of edges ordered by decreasing similarity confidence is considered. In other words, the first edge in *L*_*B*_ connects the pairs of records from *D*×D* where the attack algorithm sees the highest probability that these should be linked, and so on. As the ground truth is known in our experiments, it allows us to check how many of the pairs with maximum similarity confidence are indeed true matches, referring to a correct re-identification. For instance, assume for one attack that 8 records are correctly re-identified for the top 10 encoded records. Then the accuracy of this attack for top-10 re-identification is defined as 80%. The evaluation process of the attack is shown in Fig 4.

### Dataset

The dataset to be used is a synthetic dataset produced for record-linkage training by Eurostat and is therefore denoted as Eurostat dataset (available at: https://ec.europa.eu/eurostat/cros/content/job-training_en). It includes names, birthdays, addresses, and other attributes for 25,343 simulated people. For the experiments, the encoded database and the plaintext database are subsets of the Eurostat dataset with a specified overlap rate.

### Environment

The experiments of the attack are implemented using the Python code from [8]. Python 3.8.5 is used on an Ubuntu 20.04 server with 64-bit Intel Core i7-10750H 2.6GHz CPUs and 32 GBytes of memory. The initial attack by [8] was using Python 2.7 and running on a server with 64-bit Xeon 2.1 GHz 16-Core CPU, 512 GBytes of memory, and Ubuntu 18.04.

### Simulation parameters and outcomes

In the experiments, three different scenarios are simulated:

- In the first scenario, a random sample of 4000 records from the Eurostat database is used for encoding the resulting D*. The plaintext database *D* consists of the identical 4000 records and an additional number of randomly selected other records from Eurostat.
- In the second scenario, a sample of 4000 records according to their geographical addresses is chosen and encoded. The sample is a complete enumeration of all records within a geographical neighbourhood. This case would reflect the situation of a local registry, for example, a local health care provider. The attack database consists of the identical records and—with decreasing overlap—an increase in the number of records not contained in the 4000 records but in their geographical proximity.
- In the third scenario, two cases are considered: either the encoded database is a subset of the plaintext database or the other way around. Slightly abusing the notation, the former case is denoted as $D^{*}⊊D$ and the latter case is denoted as $D^{*}⊋D$. The larger database is also sampled randomly. This is motivated by the fact that even if the overlap rate is not high, an attacker could in principle re-identify all records contained in the smaller database. So investigating these cases may help to understand whether a low overlap rate automatically makes the attack ineffective.

The number of sampled records is chosen as it’s comparable to the original graph matching attack paper [8] and it’s common in health and medical experimental settings. For the encoding, bigrams with random hashing [13] and *s* = 15 hash functions are used. The linkage data is mapped to binary vectors with CLKs (Cryptographic Long-term Keys, [13]). The length of the bit array is *n* = 1000. For the comparison of plaintext records, we use the Dice coefficient. The attributes used as linkage data are First Name, Last Name, and Street. All parameter choices are according to [8].

As outcome measure, we used top-10, top-100, top-500 and top-1000 re-identifications. For example, a ‘top-10’ re-identification is the correspondence between one of the top matched 10 record pairs (from the encoded database and the plaintext database) to a correct pairing. For instance, there is a record pair (‘ls451gn025001’, ‘patrick’, ‘morrison’, ‘25 woodlands road’) and (‘ls451gn025001’, ‘patrick’, ‘morrison’, ‘25 woodlands road’) within the top 10 most similar pairs. There could also be a record pair within the top 10 similar pairs, but with different linkage keys. For instance, (‘isabelle’,’chapman’,’12 park road’) and (‘cameron’,‘robins’,‘20 park road’) is an incorrect pairing.

### Results

#### First scenario: 4000 randomly selected records

**Fig 5:**
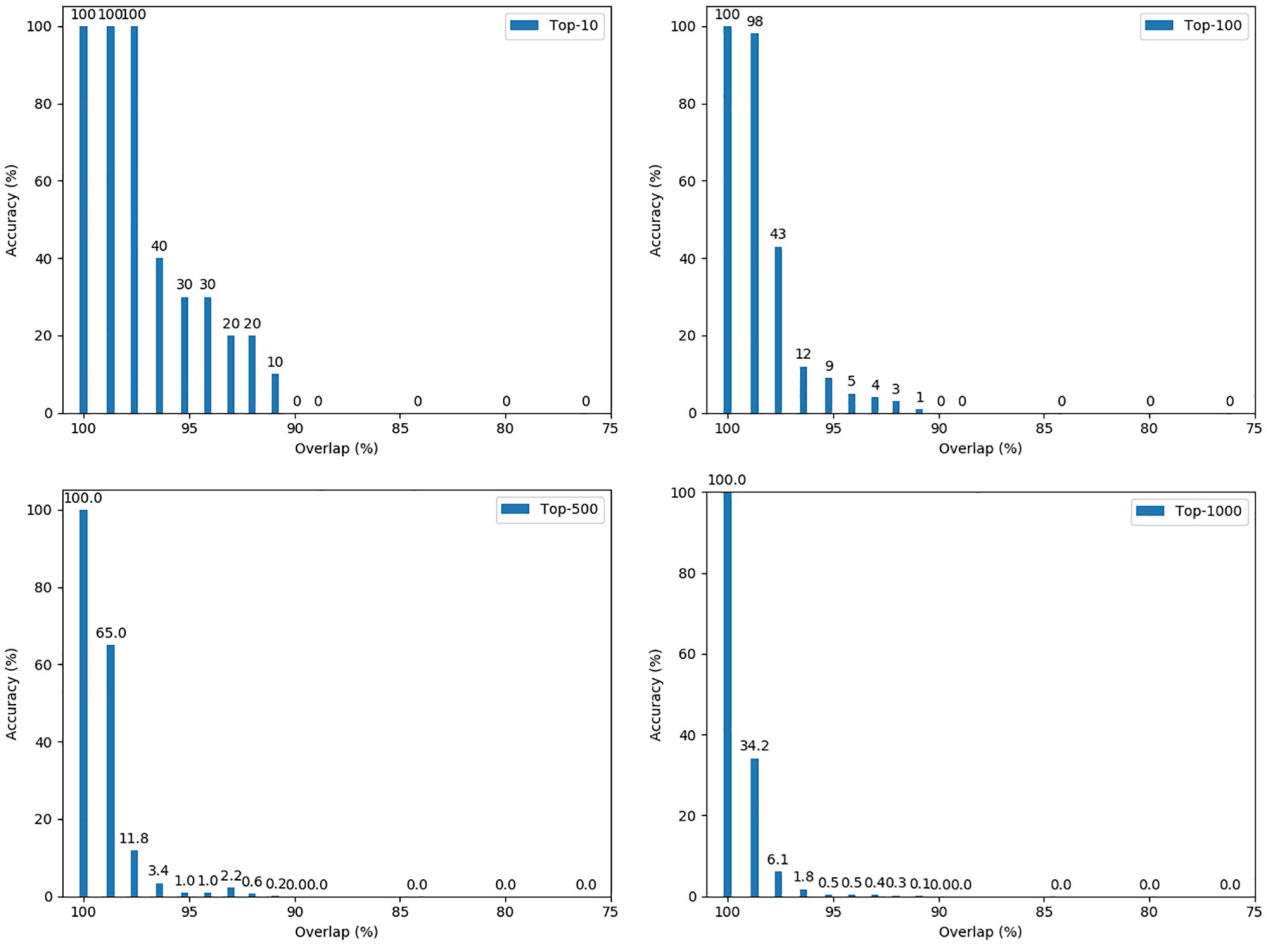
Accuracy of re-identification depending on the overlap for randomly selected records.

The results of the experiments for this scenario are shown in Fig 5. Each bar shows the maximum accuracy of five replications of the experimental parameter settings, depending on the overlap rate. In total, it’s the maximum accuracy of 400 outcomes for each parameter settings.

As one can see, the attack achieves perfect accuracy. for the graph-matching when the overlap rate is 100%. However, with decreasing overlap rate between 100% and 90%, all outcome measures decreases sharply. When the overlap falls below 90%, we observe no correct re-identifications anymore.

Moreover, we want to emphasise that in our experiments, maximum accuracy was not achieved by a single setting, but by a search given ground truth. Since so far no strategy is known for choosing at least acceptable settings for an attack, it seems doubtful if reasonable accuracy can be achieved by an attacker in practice.

#### Second scenario: 4000 non-randomly selected records

**Fig 6:**
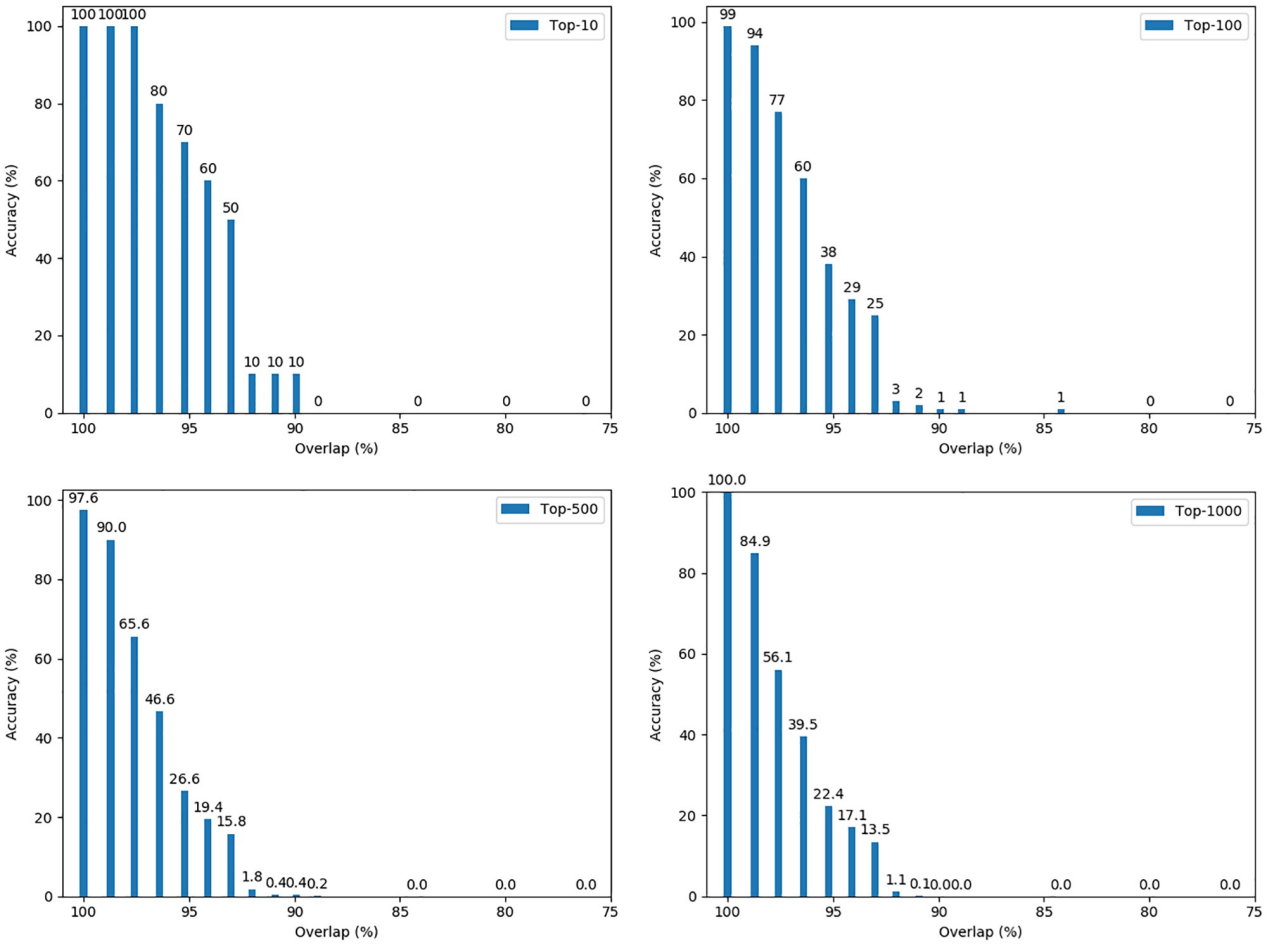
Accuracy of re-identification depending on the overlap for non-randomly selected records.

The results of the experiments for this scenario are shown in Fig 6. Compared with the first scenario, the feature is quite similar. However, there are some differences. For 98% overlap (which is the second bar from the left), the accuracy of top-10 and top-100 are nearly the same for both scenarios. For top-500 and top-1000 re-identifications, the accuracy remains high for non-randomly selected records but not for randomly selected records. Even for the experiment with 88% overlap, the attack still succeeds to some extent for the non-random records while the attack completely fails in the case of random records.

We conjecture that the reason is that people living at the same address tend to be family members. Hence their last names, as well as addresses and zip-codes, are more similar. Thus both graphs will have more edges that have high similarities than graphs using random records. Therefore, the features of the nodes will be more distinguishable, and the bi-partite graph matching task will be easier.

#### Third scenario: Proper subsets

As explained, in the third scenario we investigate the two cases $D^{*}⊊D$ and $D^{*}⊋D$. The results of high overlap rate has already been exploited in previous scenarios, therefore we only consider the case where D*, *D* has a small overlap rate. The results in Table 2 shows that the graph-matching attack never succeeds in such cases.

**Table 2:** Accuracy of re-identification for databases which form proper subsets.

	|D*|	|*D*|	*OverlapRate*	*t* = 10	*t* = 100	*t* = 500	*t* = 1000
$D^{*}⊊D$	5,000	10,000	66.7%	0	0	0	0
$D^{*}⊊D$	3,000	10,000	46.2%	0	0	0	0
$D^{*}⊊D$	1,000	10,000	18.2%	0	0	0	0
$D⊊D^{*}$	10,000	5,000	66.7%	0	0	0	0
$D⊊D^{*}$	10,000	3,000	46.2%	0	0	0	0
$D⊊D^{*}$	10,000	1,000	18.2%	0	0	0	0

## Discussion

Although the graph matching [8] is a powerful attack on PPRL schemes, at least for BF-PPRL the accuracy of the attack decreases with decreasing overlap rate. When the overlap rate is close to 100%, the attack results in highly accurate re-identifications. For all scenarios and criteria considered, the accuracy of re-identification decreases sharply when the overlap rate gets smaller.

This seems to be inevitable. In spite of the fact that all information in the encoded database is contained in the plaintext database in our experimental settings, the differences of the feature matrices increase as the overlap rate between the two databases decreases. Thus the difficulty of the bi-partite graph matching problem is amplified and the ability to correctly re-identify records is diminished.

Although we can not assert with mathematical certainty that the attack will be unsuccessful for all lower overlap rates, the accuracy will approach random agreement of record-pairs with decreasing overlap rates.

### Preventing the attack by decreasing overlap using fake record insertion

As previously shown, the overlap rate is essential for an evaluation of the success probability of the attack. While we claim that an overlap rate close to 100% is unlikely to happen in practice, such cases may exist. Given the high effectivity of graph matching attacks in such cases, there is the need for appropriate countermeasures.

Recall that our experiments indicate that when the overlap rate falls below 90%, the success probability of the attack quickly tends to zero. This even holds for cases where one database is a proper subset of the other. Such a situation can be achieved by the insertion of fake records, i.e., records that do not exist in the actual database. Note that fake records cannot be detected by an attacker if these have been inserted into the encoded database.

More precisely, assume the case that the plaintext database *D* and the encoded database D* which are equal, meaning an overlap rate of 100%. Before the encoded database is handed out, *α*⋅|D*| fake records are inserted into the encoded database *D*, increasing its cardinality to (1 + *α*)|D*|. A simple calculation shows that the overlap rate between the encoded database with the fake records and plaintext database is at most

$$OverlapRate=\frac{1}{1+\alpha }.$$

We study the effect of inserting fake records on the graph matching attack by comparing its accuracy before and after the insertion of fake records. In this experiment, the overlap rate of two databases is 100% before insertion and 80% after insertion.

**Fig 7:**
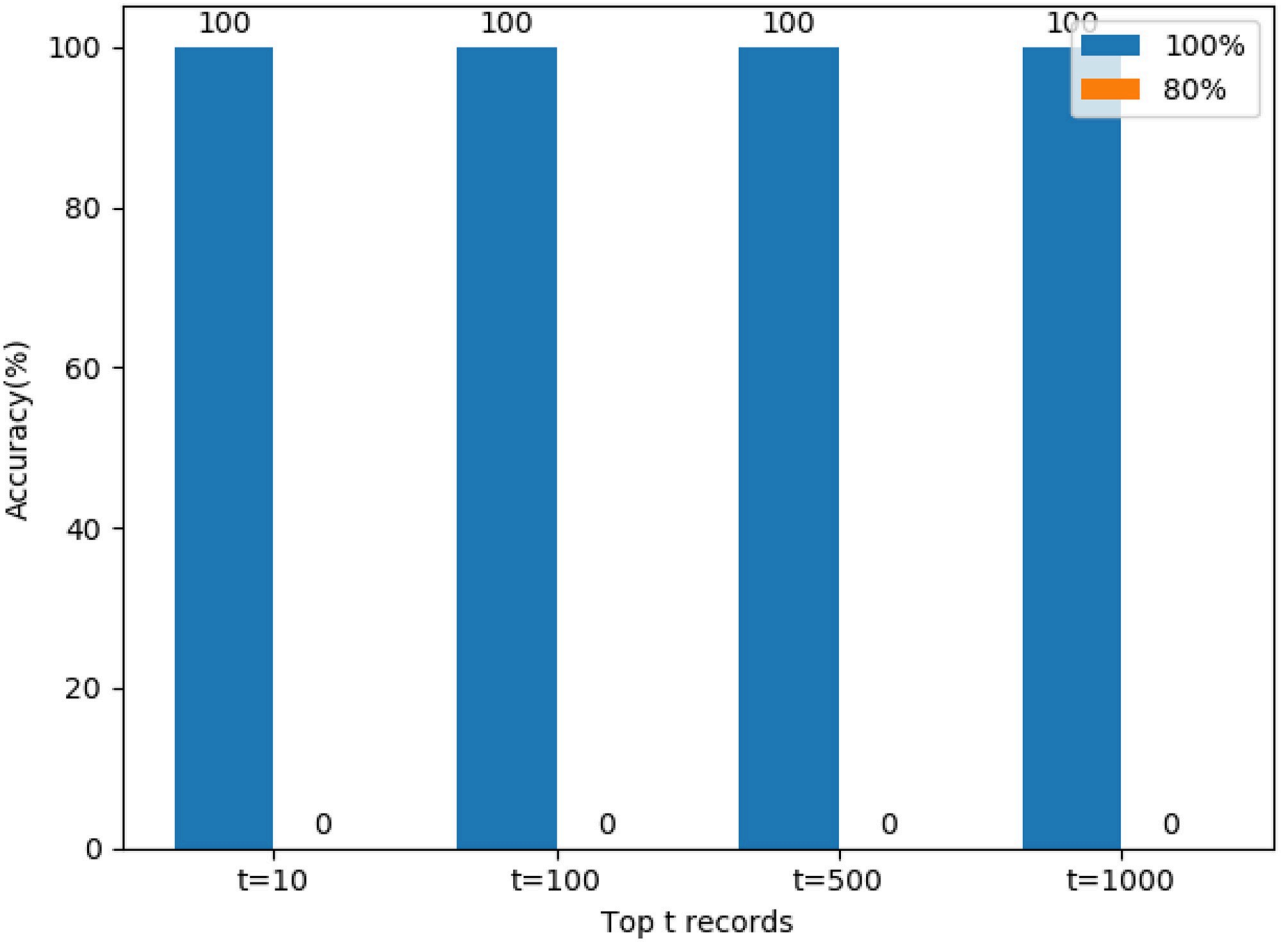
Accuracy of re-identification before and after 0.25 fake injections success rate drops from 100% to 0%.

Fig 7 shows the effect of inserting fake records into the encoded database and making the overlap rate *OverlapRate* = 80%, i.e. setting *α* = 0.25. Regardless whether the top 10 or the top 1000 records are used for comparison, the success rate drops from 100% to 0% after the insertion of *α* = 25% fake records. Therefore, we consider fake records as an efficient countermeasure for preventing graph-matching attacks. Note that in [14], the authors likewise suggested the use of fake records. However, their goal was to modify the frequency distribution to thwart frequency attacks. Our experiments show the effectivity of this approach against a different type of attack.

## Conclusion

We conducted a study on the accuracy of the graph matching attack on privacy-preserving record linkage (PPRL). Although the graph matching attack can re-identify sensitive record values in an encoded database using a plaintext database, specific conditions rarely given in practice are necessary for a successful attack. The re-identification is almost perfect when the plaintext database has a high overlap with the encoded database. However, the accuracy of the attack decreases sharply when the overlap rate of the encoded database and plaintext database decreases. The overlap can be reduced by using fake records to make the graph matching attack difficult in practice. Therefore, the theoretical existence of the graph matching attacks do not prohibit the application of BF-PPRL under jurisdictions such as the European Data Protection Regulation, requiring not absolute anonymity for research databases but demands an irrational effort for already illegal re-identification attempts [1].

We see several directions for possible future work. For instance, the experiments reported in this paper focus on BF-PPRL because they are widely used in practice. It could be interesting to extend the studies to other PPRL methods. Moreover, it remains open to conduct theoretical analysis on the relation between the accuracy of graph matching attacks and the overlap rate. If this relation would be better understood, the impact of the attack in practice could be better evaluated and more effective countermeasures could be designed.

## Supporting information

S1 File (ZIP)
